# A randomized controlled study of a combination of internal and external treatments for albumin paclitaxel-related peripheral neurotoxicity: A randomized controlled: A study protocol

**DOI:** 10.1097/MD.0000000000032252

**Published:** 2022-12-23

**Authors:** Xiaoli Li, Qimeng Sun, Hao Ding, Shulan Hao, Yonglin Lan, Likun Liu, Ruimin Wang

**Affiliations:** a Department of Oncology, Shanxi Province Hospital of Traditional Chinese Medicine, Taiyuan, China.

**Keywords:** bloodletting therapy, chemotherapy-induced peripheral neuropathy, Huangqi Guizhi Wuwu decoction, nab-paclitaxel, quality of life

## Abstract

Albumin-bound paclitaxel (nab-PTX), a novel paclitaxel preparation, has been found to successfully blocks tumor progression in breast and lung cancer. However, at the same time of as clinical application, neurotoxicity caused by nab-PTX has become the main factor limiting the clinical application of nab-PTX, which seriously affects the quality of life of patients and increases their psychological or financial burden. In clinical applications, JHGWD combined with bloodletting therapy at the end of the extremities has a positive effect on neurotoxic symptoms such as numbness, pain, and weakness of the hands and feet caused by nab-PTX. In a single-arm experiment, it was also found that the immediate effective rate of exsanguination therapy was as high as 70%, and when combined with oral Chinese medicine treatment, it further improved the efficacy. Therefore, a randomized controlled trial (RCT) was designed to further evaluate the efficacy and safety of this treatment.

**Methods:**

This RCT will be conducted at the Shanxi Provincial Hospital of Traditional Chinese Medicine. A total of 120 patients with Nab-PTX chemotherapy-induced neurotoxicity will be recruited. Treatment groups will be categorized into herbs alone group, bloodletting treatment alone group, and herbs combined with bloodletting group. Blank control was used. The primary outcome will be the EORTC QLQ-CIPN20 scale of the included patients, and the secondary outcomes will include EMG, peripheral neurotoxicity symptom score, NCI-CTCAE5.0 peripheral neurotoxicity grade, and WHO anti-tumor drug peripheral neurotoxicity grade. Adverse reactions will be recorded throughout the process. All data in this RCT will be analyzed by SPSS 26.0 software.

**Discussion:**

The results of this RCT will contribute to treating PIPN, relieving the neurotoxic symptoms, and improving the quality of life of patients. Finally, the RCT results will be published in a relevant academic journal on completion of the trial.

**Trial registration:**

ChiCTR2200060217(May22,2022)

## 1. Introduction

Albumin-bound paclitaxel (nab-PTX) is widely used in the treatment of breast^[1]^ and lung cancers^[2]^ due to its excellent therapeutic effects. However, the high incidence of chemotherapy-induced neurotoxicity, which impairs the patients’ quality of life,^[3]^ affects the efficacy of chemotherapy and therapeutic interventions. The most common manifestation of neurotoxicity is numbness in the hands and feet, which persists many months or even years after the completion of chemotherapy. To the best of our understanding, there is no unified standard for the assessment and diagnosis of chemotherapy-induced peripheral neuropathy (CIPN),^[4]^ this far, and its mechanism is still unclear. Higher doses of nab-PTX and weekly chemotherapy are more likely to cause numbness in the extremities of the patients,^[5]^ and the presence of underlying diseases such as diabetes mellitus, cerebral infarction, and cervical spondylosis negatively interferes with patient treatment. However, the existing treatment methods are limited and ineffective. At present, the measures to prevent the neurotoxicity of purple shirt include surgical glove compression therapy^[6]^ and the ice glove method.^[7]^ Akiko H et al^[8]^ conducted a self-controlled study of prophylactic cryotherapy on 36 breast cancer patients who had received paclitaxel chemotherapy. The study results revealed patients who did not undergo prophylactic cryotherapy experienced significantly decreased touch and temperature sensation in their hands compared with those who were treated with paclitaxel chemotherapy alone. Because of inconvenient operation and poor patient compliance, it has not been widely used in clinical practice. According to the literature, duloxetine has proven to be an effective medicine that cures neurotoxicity.^[4,9]^ It belongs to the class of serotonin-norepinephrine reuptake inhibitors, which are generally used in the treatment of generalized anxiety disorder and depression. Although duloxetine significantly alleviates neuropathic pain,^[10]^ which is just one of many neurotoxicity symptoms,^[11]^ it does not help in the treatment of numbness effectively. Moreover, it has many side effects, including gastrointestinal reactions such as nausea and vomiting, as well as neurological disorders such as insomnia, anxiety, drowsiness, vertigo, and tremor.^[12]^ Therefore, duloxetine is rarely used in clinical practice. Hence, finding new and well-tolerated treatments for paclitaxel induced peripheral neuropathy (PIPN) remains an ongoing challenge for oncologists.

Traditional Chinese medicine plays a unique role in the comprehensive treatment of cancer, including reducing the toxic side effects related to cancer treatment, prolonging the survival of patients, and improving the quality of life of patients, which makes it an indispensable treatment method for cancer patients. Moreover, it is a simple and cost-effective treatment option with low toxicity and superior effect and provides a new treatment mode for tumor patients. There have been some studies on the treatment of chemotherapy-related neurotoxicity with traditional Chinese medicine, mainly including herbal infusion for hands and feet therapy,^[13]^ acupuncture, and appropriate exercise,^[14,15]^ which can improve neurotoxic symptoms. However, there is a lack of high-quality and satisfactory evidence,^[16]^ which is insufficient to meet the clinical needs. In our study, we have found that the use of bloodletting therapy in clinical practice can enhance the efficacy of traditional Chinese medicine (TCM) treatment, with rapid onset and lasting effects combined with the oral administration of TCM.

In ancient Chinese books, Huangqi Guizhi WuWu decoction (HGWD) is a classic formulation that is used for the treatment of numbness and sensory dysfunction of the limbs. An oral Chinese herbal medicine (CHM) known as the Jiawei Huangqi Guizhi Wuwu decoction, or JHGWD, is derived from this formulation with the addition of 4 more herbs to increase its efficacy in treating neurotoxicity. Cao Peng’s team^[17]^ extracted AC591 from HGWD and confirmed that AC591 could reduce the occurrence of chemotherapy-induced neuropathy by establishing a rat model of peripheral neurotoxicity. Ancient Chinese medicine books record that bleeding from one end of the limb can cure numbness of the hand and foot before the occurrence of a stroke. In clinical practice, bloodletting from the tips of the hands and feet is found to be a suitable treatment option for patients with PIPN. To this end, a single-arm experiment was conducted: Bloodletting therapy with or without JHGWD. This experiment included 44 patients with hand and foot numbness after using nab-PTX. In a 2-week observation period, it was found that the continuous curative effect of traditional Chinese medicine combined with bleeding was 100%, and the autonomic scores of peripheral nerve symptoms of patients were decreased. Moreover, the operation was simple, fast, and convenient for wide application. The purpose of this study is to verify the efficacy of JHGWD combined with bloodletting therapy in the treatment of PIPN.

## 2. Materials and Methods

### 2.1. Study design

**Figure 1.**
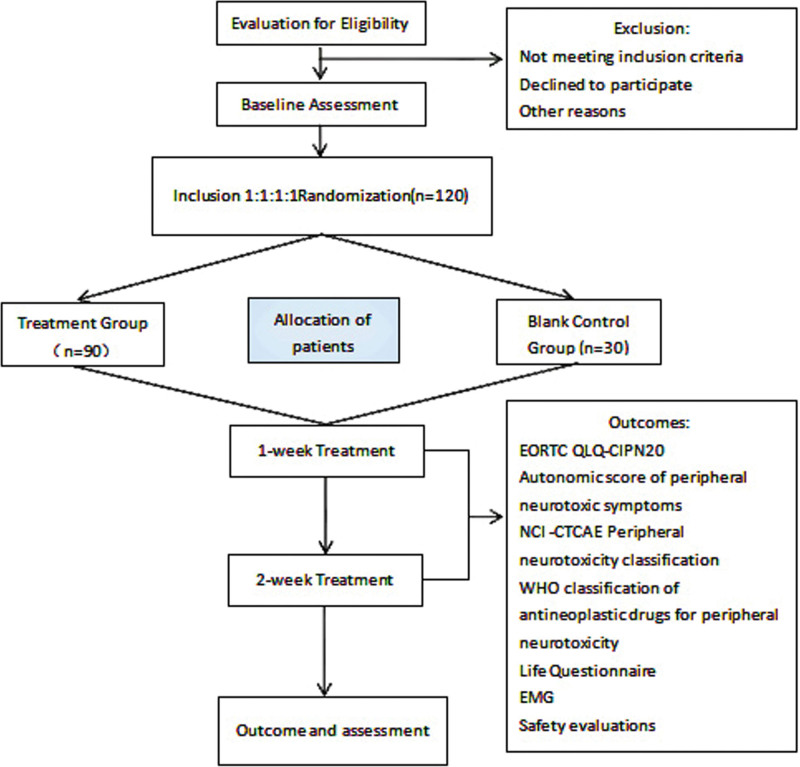
Study flowchart.

This is a prospective, randomized controlled trial (RCT) that combines bloodletting therapy with JHGWD. A parallel group of 120 patients with breast or lung cancer in the presence of PIPN was recruited. The included patients were planned to be divided into 4 groups: 3 treatment groups (JHGWD alone, bloodletting therapy alone, bloodletting therapy combined with JHGWD), and a blank control group. The comparison of the efficacy of the treatment before and after 2 weeks was analyzed (Fig. 1).

### 2.2. Ethics and registration

This RCT has been approved by the Human Research Ethics Committee of Shanxi Province Hospital of Traditional Chinese medicine (2022-07002) and has been registered with the Chinese Clinical Trial Registry (ChiCTR2200060217). Participants who sign the informed consent form will be enrolled in the RCT, and they can withdraw from this study at any time.

### 2.3. Patients

For the RCT, patients will be recruited from the Shanxi Province Hospital of Traditional Chinese Medicine. Adequate sample sizes will be obtained through hospital departments and online platforms, and the inclusion criteria will be verified. Those who have relatively complete medical records, and have signed informed consent.

#### 2.3..1. Inclusion criteria.

① Patients with the diagnosis of lung or breast cancer that was pathologically and cytologically confirmed;
② Age of the patients should be above 18 years and below 80 years, regardless of gender;
③ Patients with a KPS score of ≥ 60 and an excepted survival of at least 3 months;
④ Patients who have developed peripheral neurotoxicity after receiving albumin-bound
paclitaxel treatment;
⑤ Patients with no major visceral organ dysfunction and no cardiovascular or cerebrovascular complications.
⑥ Patients with intact skin on all 4 limbs;
⑦ Patients who can completely understand the situation of this clinical trial requirement voluntarily accept the TCM treatment, and voluntarily answer the questionnaire for good compliance;

#### 2.3..2. Exclusion criteria.

① Patients with reduced platelets prone to bleeding;
② Patients who do not meet the above inclusion criteria;
③ Patients who have symptoms related to nerve compression due to brain metastases or who have peripheral nerve damage due to tumor compression;
④ Patients with other previous peripheral neuropathies (e.g., neurological diseases, orthopedic related diseases, and peripheral neuropathies due to circulatory disorders);
⑤ Patients with a history of allergy to drug exposure or the presence of skin lesions on the hands and feet (e.g., eczema);
⑥ Patients who are unable to cooperate with the treatment;
⑦ Pregnant or breastfeeding women;
⑧ Patients who are participating in other clinical trials currently or within 4 weeks;
⑨ Allergic individuals who are susceptible to hypersensitivity to the drug.

#### 2.3..3. Shedding criteria.

① Patients who have experienced serious adverse events, such as infection at the site of bloodletting, and who are not suitable for further observation;
② Patients with incomplete information and unwillingness to undergo return visits, etc, which affects the assessment of treatment;
③ Patients who have poor compliance and do not complete treatment as required;
④ Patients who do not wish to continue treatment and request to withdraw from the trial;
⑤ Patients with changes in their health conditions that affect or interfere with this study.

### 2.4. Sample size

Referring to the results of a small sample of clinical trials conducted by our team in a previous single-arm trial, the treatment group was effective at 70%. According to PASS15 software, the estimated shedding rate is 20%, requiring 30 participants per group.

### 2.5. Randomization

Sequences will be generated and assigned by the data managers. The selected participants will be randomly divided into three treatment groups and a blank control group in a 1:1:1:1 ratio according to the random number table method.

### 2.6. Intervention measures

The treatment groups are divided into herbal medicine alone, bloodletting alone, and herbal medicine combined with bloodletting groups. The blank control group is not subject to intervention at any point of time. The treatment period is 2 weeks through face-to-face consultation and telephone follow-up. Electromyography was performed on days 1 and 14 of treatment, bloodletting was performed on days 1 and 7 of treatment, and Chinese herbal medicine was given orally for 14 d continuously. All patients avoided cold drinks and contact with cold objects and cold water on the arms during the observation period. All the selected participants will be supervised by an experienced oncologist.

### 2.7. Outcomes

#### 2.7.1. Primary outcomes.

EORTC QLQ-CIPN20 scale.

#### 2.7.2. Secondary outcomes.

1. EMG nerve conduction velocity;
2. Peripheral neurotoxicity symptom score (patients rate themselves according to the degree of numbness in their hands and feet on a scale of 0–10, with higher scores indicating severe numbness and lower scores indicating less numbness.);
3. NCI-CTCAE5.0 peripheral neurotoxicity grade;
4. WHO antitumor drug peripheral neurotoxicity grade.

### 2.8. Safety evaluation

During the entire period of this RCT, each adverse event will be recorded by a dedicated treating physician in detail.

### 2.9. Data management and quality control

Any changes to the protocol should be approved by the Ethics Committee of Shanxi Province Hospital of Traditional Chinese Medicine. All data from patients will be recorded in a CRF form by professional researchers. Any information on patients is confidential.

### 2.10. Baseline assessments

Sociodemographic data are planned to be collected from the included patients, and also the data on duration of disease and previous herbal medicine treatments.

### 2.11. Statistical analysis

The data analysis setting will follow intent-to-treat principles. Data will be statistically analyzed using SPSS 26.0 software. The Chi-squared test will be used for enumeration data, and mean ± standard will be used for measurement data. The difference between the treatment group and the control group will use log-rank. Any *P* value less than .5 is considered as a statistically significant difference. The differences of outcomes will be discussed in subgroup analysis.

## 3. Discussions

PIPN has an effect on patients’ life standards. In the worst-case scenario, it can lead to a delay or even termination of chemotherapy. Acute PIPN will slowly disappear after the completion of chemotherapy, in a number of cases, it will last for months or even years. In some cases, PIPN can emerge slowly after finishing chemotherapy, which is a phenomenon known as“coasting.”^[18]^ Studies have shown that level of PIPN can happen when the paclitaxel accumulates 100 to 300 mg/m^2^ in the body, and it accumulations of more than 1000 mg/m^2^ which can lead to severe PIPN.^[19]^ The dose of nab-PTX is 260 mg/m^2^, and the cumulative dose after the completion of 4 cycles of chemotherapy is over 1000 mg/m^2^. Therefore, nab-PTX is more likely to cause neurotoxicity, and this issue needs to be resolved.^[20]^ Related studies have shown that paclitaxel caused chronic neurotoxicity, and targets the dorsal root ganglion (DRG) neurons, and after the administration of paclitaxel, its concentration was found to be higher in DRG cells than that in other peripheral nerves.^[21]^ Arianna Scuetri et al showed that paclitaxel neurotoxicity is likely to adversely affect the architecture of microtubules in DRG axons,^[22]^ thereby affecting axon transport and ultimately reducing the axon growth rate. Nicole’s study suggested that the nerve pain caused by paclitaxel may be associated with TRPV4-mediated hyperalgesia and it increased the speed of receptor conduction, which relies on tyrosine kinase signaling pathways.^[23]^ At present, studies have found that proinflammatory cytokines and chemotactic factors are related to the occurrence of neuropathic pain, and they can increase pain by inhibiting inflammatory pathways.^[24]^ For example, Segat GC et al found that β-caryophyllene can prevent the raise of Iba-1 and reduce the activation of inflammatory factors in the mouse model.^[25]^ In breast cancer studies, serum neurofilament light chain levels^[26]^ may be used for predicting the severity of PIPN. In conclusion, there are many mechanisms for the occurrence of PIPN, but they are limited to theory only. To the best of our knowledge, there are no symptomatic drugs for targets related to chemotherapy-induced peripheral neurotoxicity as there are even fewer studies on peripheral neurotoxicity due to albumin-bound paclitaxel and even less information on the corresponding drug treatment. The combined internal and external treatment method of Chinese medicine can fill the gap.

The treatment of PIPN in Chinese medicine requires evidence-based treatment, including general treatment with an oral decoction and local treatment with bloodletting therapy. Bloodletting therapy combined with JHGWD can further improve the efficacy of CHM in the treatment of post-chemotherapy neurotoxicity.

Professor Li-Kun Liu, a professional Chinese medicine doctor, has proven that the formulation of JHGWD is effective in treating PIPN in clinical practice. The decoction is composed of 10 herbs: Huangqi, Guizhi, Baishao, Danggui, Jixueteng, Laoguancao, Xixin, Shuizhi, Shengjiang, and Dazao(All the above herbs are provided by the herbal pharmacy of Shanxi Provincial Hospital of Traditional Chinese Medicine). These herbs are multifunctional because of their antioxidant and neuroprotective properties^[27]^ and can increase the speed of nerve conduction.^[28]^

The results of this RCT have clinical practice implications for the treatment of PIPN with the addition of JHGWD combined with bloodletting therapy. Our primary focus is to conduct a high-quality RCT that utilizes validated evaluation measures for not only reducing neurotoxic symptoms and improving patients’ quality of life, but also focusing on exploring the further assessment of the effectiveness and safety of the treatment. Finally, the results from this RCT will be published in a relevant academic journal on completion of the trial.

## Author contributions

**Conceptualization:** Xiaoli Li, Li-Kun Liu.

**Data curation:** Qimeng Sun, Yonglin Lan,Ruimin Wang.

**Methodology:** Hao Ding.

**Supervision:** Likun Liu,Shulan Hao,Xiaoli Li

**Writing – original draft:** Xiaoli Li,Qimeng Sun

**Writing – review & editing:** Xiaoli Li,Qimeng Sun
